# Minimizing scattering-induced phase errors in differential interference contrast microscopy

**DOI:** 10.1117/1.JBO.25.12.123703

**Published:** 2020-12-14

**Authors:** Wataru Takano, Shuhei Shibata, Nathan Hagen, Masaru Matsuda, Yukitoshi Otani

**Affiliations:** a Utsunomiya University, Department of Optical Engineering, Tochigi, Japan; b Utsunomiya University, Center for Optical Research and Education, Tochigi, Japan; c Utsunomiya University, Center for Industry-University Innovation Support, Tochigi, Japan; d Utsunomiya University, Center for Bioscience Research and Education, Tochigi, Japan

**Keywords:** differential interference contrast microscope, polarization camera, quarter-wave plate, real-time phase measurement

## Abstract

**Significance:** Differential interference contrast (DIC) microscopes allow noninvasive *in vivo* observation of transparent microstructures in tissue without the use of fluorescent dyes or genetic modification. We show how to modify a DIC microscope to measure the sample phase distribution accurately and in real-time even deep inside sample tissue.

**Aim:** Our aim is to improve the DIC microscope’s phase measurement to remove the phase bias that occurs in the presence of strong scattering.

**Approach:** A quarter-wave plate was added in front of the polarization camera, allowing a modified phase calculation to incorporate all four polarization orientation angles (0 deg, 45 deg, 90 deg, and 135 deg) captured simultaneously by the polarization camera, followed by deconvolution.

**Results:** We confirm that the proposed method reduces phase measurement error in the presence of scattering and demonstrate the method using *in vivo* imaging of a beating heart inside a medaka egg and the whole-body blood circulation in a young medaka fish.

**Conclusions:** Modifying a polarization-camera DIC microscope with a quarter-wave plate allows users to image deep inside samples without phase bias due to scattering effects.

## 1 Introduction

Noninvasive *in vivo* observation of transparent microstructures such as cells is important for biology and medicine. Differential interference contrast (DIC) microscopes make it possible to observe a transparent sample without fluorescent dyes or genetic modification by converting sample phase gradients into intensity variations through shearing interferometry.^1^ Although this renders phase gradients visible, quantifying the sample’s phase distribution requires collecting multiple images. Many researchers have proposed methods for quantitative phase measurement using transmission DIC microscopy^2^^,^^3^ and surface profile measurement using reflection DIC microscopy.^4^^–^^8^ These methods are difficult to use for living samples because they require taking multiple images while rotating the polarization analyzer to multiple angles. For *in vivo* measurement, researchers have developed techniques where the input and output of the Nomarski set up can be electronically modulated, demonstrating speeds of 1 frame in 12 s, although higher speeds are likely possible.^9^

In addition to DIC microscopy, other phase imaging techniques exist and have been adapted for video detection. Zernike phase contrast microscopy (PCM) has been adapted with color multiplexing to collect video-rate phase images.^10^ Although PCM has a simpler hardware set up than DIC and is not subject to error from object birefringence, it typically provides lower axial resolution (optical sectioning) and produces haloes around phase objects.^11^^,^^12^ Ptychography and digital holographic microscopy are more recent methods that can also provide video-rate phase imaging.^13^^,^^14^

In our previous paper,^15^ we quantified the phase distribution in real time for a DIC microscope using a polarization camera—a camera for which a micropolarizer array has been attached to the detector array.^16^^–^^18^ A polarization camera can detect the intensity at four polarization orientation angles (0 deg, 45 deg, 90 deg, and 135 deg) in a snapshot, so that detection speeds are limited only by the frame rate of the detector. We previously used only two intensities detected using the pixels oriented at 45 deg and 135 deg of the micropolarizer array. That is we used only half the light intensity detected by the detector. In such a set up, the intensities detected using the pixels oriented at 0 deg and 90 deg do not produce interference because these axes are aligned to the shearing direction. We show below that using only two of the four micropolarizer orientations decreases the quantitative accuracy of phase measurement with this method in the presence of scattering. We show that adding a quarter-wave plate in front of the polarization camera and using the full light intensity at all four polarization orientation angles (0 deg, 45 deg, 90 deg, and 135 deg) improves the phase measurement accuracy, especially in the presence of scattering.

## 2 Quantification Method of Phase Distribution

**Fig. 1:**
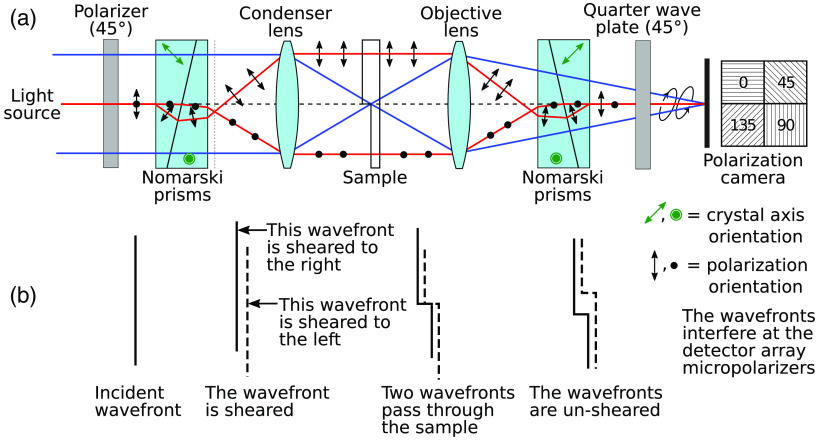
(a) The optical system of the DIC microscope using a polarization camera, with a quarter-wave plate added after the second Nomarski prism pair. (b) The change of the wavefront as light passes through the system.

A DIC microscope modifies a microscope’s optical layout by adding polarization optics to both the illumination and imaging sides as shown in Fig. 1. The resulting system utilizes lateral shearing interference between the two orthogonally polarized beams produced by a pair of Nomarski prisms and passing through slightly shifted paths (shifted by the shear distance Δx) through the sample. The two beams are then combined by a second pair of Nomarski prisms that cancel the shear. The resulting interferogram observed at the imaging plane encodes the spatial gradient of the object phase along the shear direction.

The two orthogonal beams are expressed as

U1(x)=α1 exp[iθ(x+12Δx)−iωt]e^x,U2(x)=α2 exp[iθ(x−12Δx)−iωt]e^y, (1)

where x is the one-dimensional spatial coordinate, Δx is the shear distance, $\alpha_{1}$ and $\alpha_{2}$ are the amplitudes of the two beams, θ(x) is the phase of the beams, ω is the angular frequency, t is the time, and e^x and e^y are the unit vectors along the x and y axes.

When these two beams pass through a polarizer oriented at angle φ, they are forced to interfere, and the resulting time-average intensity is obtained as

I(x,φ)=[α12 cos2 φ+α22 sin2 φ+2α1α2 cos φ sin φ cos(Δθ+2β)]e^φ, (2)

where $\Delta \theta \approx \theta (x+\frac{1}{2}\Delta x)-\theta (x-\frac{1}{2}\Delta x)$, β is the bias phase, and e^φ is a unit orientation vector, pointing along the transmission axis of the polarizer. For the two beams sheared by the Nomarski prisms

$$\frac{\Delta \theta }{\Delta x}=\frac{\theta (x+\frac{1}{2}\Delta x)-\theta (x-\frac{1}{2}\Delta x)}{\Delta x}\;\approx \frac{\left[\theta (x)+\frac{1}{2}\Delta x\cdot \frac{d\theta }{dx}]-[\theta (x)-\frac{1}{2}\Delta x\cdot \frac{d\theta }{dx}\right]}{\Delta x}=\frac{d}{dx}\theta (x),$$ (3)

so that the phase gradient dθ/dx detected by the DIC microscope is given by Δθ, the phase difference induced by the sample between the two sheared beams, and Δx is the shear distance. We adjust the DIC microscope by sliding the second pair of Nomarski prisms so that the retardation along the shear direction (i.e., the bias phase) becomes 2β=π/2. In this configuration, small changes in the phase gradient cause the largest variation in intensity, so that the system has maximum phase sensitivity.

Figure 1 shows our proposed optical layout of a DIC microscope using a polarization camera in which we have added a quarter-wave plate oriented at 45 deg between the second pair of Nomarski prisms and the detector array. With this layout, the two orthogonally polarized beams produced by the Nomarski prisms are converted to left and right circular polarization by the waveplate. The polarization camera then detects the light intensity at each polarizer azimuthal angle (0 deg, 45 deg, 90 deg, and 135 deg). Although the intensities detected using the pixels oriented at 0 deg and 90 deg of the micropolarizer array do not produce interference in the conventional method, all four orientation angles sense the interference in our proposed method.

The four-phase-shift method is an algorithm for calculating the relative phase of a fringe pattern from four images taken at phase shifts 0 deg, 45 deg, 90 deg, and 135 deg. This relative phase corresponds to the spatial phase difference Δθ across the sample, as given by^2^^,^^19^^,^^20^

$$\Delta \theta (x)=\tan^{-1}\{\frac{I(x,135\;\deg)-I(x,45\;\deg)}{I(x,0\;\deg)-I(x,90\;\deg)}\},$$ (4)

where I(x,φ) is the intensity detected by a pixel behind a micropolarizer oriented at angle φ. Equation (4) holds true for all nonpolarizing samples, so that the imaged sample need not be a pure phase object (i.e., it can have nonzero extinction). The phase difference can be measured even for an attenuating medium in the four-phase-shift method because the effect of amplitude does not remain in the calculation result. The spatial phase gradient dθ/dx detected by the DIC microscope is the phase difference Δθ induced by the sample divided by the shear distance Δx

$$\frac{d}{dx}\theta (x)\approx \frac{\Delta \theta (x)}{\Delta x}=\frac{1}{\Delta x}\;\tan^{-1}\{\frac{I(x,135\;\deg)-I(x,45\;\deg)}{I(x,0\;\deg)-I(x,90\;\deg)}\}.$$ (5)

The shear distance must, therefore, be known in order to accurately quantify the phase gradient, but the shear distance is generally not disclosed by DIC microscope vendors. Our estimate follows a technique described by Shribak^21^ in which the shear distance is estimated by first measuring the shear angle, from which we use the known objective lens focal length and simple trigonometry to compute the shear distance, giving Δx∼650  μm for the Nomarski prism in our system. The shear angle was obtained by removing one pair of Nomarski prisms and transmitting a laser through them, while observing the pair of beams produced at a screen far away and calculating the splitting angle.

Following the approach of Munster et al.,^22^ we can express any phase gradient

$$\frac{\Delta \theta (x)}{\Delta x}=\frac{\theta (x+\frac{1}{2}\Delta x)-\theta (x-\frac{1}{2}\Delta x)}{\Delta x}$$ (6)

as a convolution

$$\frac{\Delta \theta (x)}{\Delta x}=\frac{g(x)⊗\theta (x)}{\Delta x}$$ (7)

of the sample phase function θ(x) with the kernel

$$g(x)=\delta (x+\frac{1}{2}\Delta x)-\delta (x-\frac{1}{2}\Delta x),$$ (8)

where δ(x) is the Dirac delta function. The spatial domain Fourier transform of Eq. (7) is expressed as

$$\frac{\Delta \Theta (u)}{\Delta x}=\frac{G(u)\Theta (u)}{\Delta x},$$ (9)

where u is the spatial frequency, and

G(u)=−i sin(2πuΔx). (10)

The obvious thing to try is $\Theta (u)=G^{-1}(u)\Delta \Theta (u)$, but $G^{-1}(u)$ is undefined where G(u) is near zero. In order to regularize the inverse, we use a Wiener filter $W_{0}(u)$

$$W_{0}(u)=\frac{G^{*}(u)}{{\left|G(u)\right|}^{2}+{\left[\mathrm{SNR}(u)\right]}^{-1}}.$$ (11)

The sample phase estimate in the Fourier domain is given by

Θ^(u)Δx=W(u)·ΔΘ(u)Δx  , (12)

and SNR(u) is the spectral distribution of the signal-to-noise ratio. Munster et al. (1997) approximated the SNR as a Gaussian

$$\mathrm{SNR}(u)=s\cdot \exp[\frac{-0.5u^{2}}{\sigma^{2}}],$$ (13)

where s is the maximum SNR(u).

**Fig. 2:**
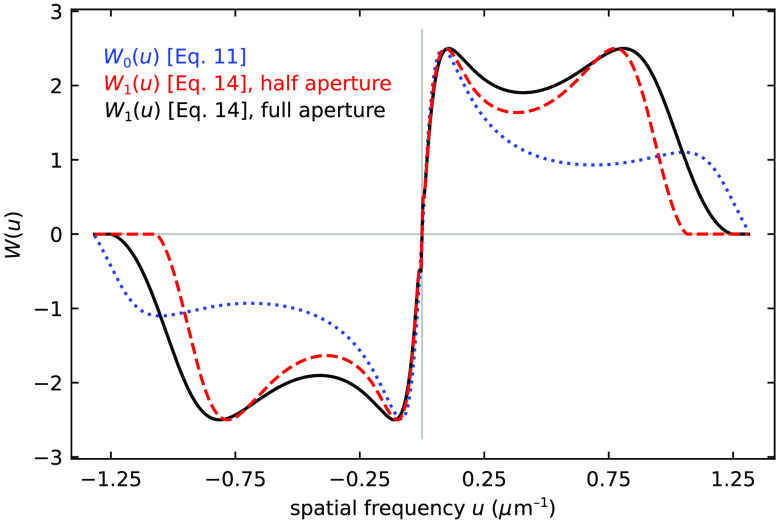
(Blue dotted curve) The regularized inverse $W_{0}(u)$ of DIC microscope without incorporating the objective lens’ OTF according to Munster et al.’s approach.^22^ (Red dashed curve.) The regularized inverse $W_{1}(u)$ of the our DIC microscope with 20× objective lens; (black curve) $W_{1}(u)$ for the same conditions at maximum aperture.

Figure 2 shows the regularized inverse $W_{0}(u)$ when s is 25, σ is 0.6, and G(u)=−i sin(1.17πuΔx), producing a function that has two peaks near 0.05 and $1.1\;(\mu m^{-1})$. This function does not yet take into account the objective lens optical transfer function (OTF). The regularized inverse system transfer function incorporating the objective lens OTF (also shown in Fig. 2) is

$$W_{1}(u)=\frac{{\left[G(u)\cdot \mathrm{OTF}(u)\right]}^{*}}{{\left|G(u)\cdot \mathrm{OTF}(u)\right|}^{2}+{\left[\mathrm{SNR}(u)\right]}^{-1}}.$$ (14)

This function has two peaks near 0.08 and $0.80\;(\mu m^{-1})$ and is zero for $u>1.25\;(\mu m^{-1})$ at maximum aperture and for $u>1.07\;(\mu m^{-1})$ at half aperture.

The sample phase θ(x) can be obtained by deconvolution using

$$\theta (x)=F^{-1}[W_{1}(u)F\{\frac{\Delta \theta (x)}{\Delta x}\}],$$ (15)

where F{ } and $F^{-1}[\;]$ are the Fourier and inverse Fourier transform, $W_{1}(u)$ is the Wiener-filtered inverse transfer function of the DIC microscope system, and Δx is the shear distance given in units of pixels.

When the phase distribution is measured using four different pixel positions on the polarization camera, the spatial resolution of the sample is worsened by a factor of 2 in each of the two spatial directions. In order to prevent the reduced spatial sampling from creating aliasing artifacts, we defocus the image so that it is spatially bandlimited to half the Nyquist limit and employ an interpolation method developed by Tyo et al.^23^ for which aliasing artifacts are zero for properly bandlimited images.^15^ In order to simplify the description, we expand from one dimension (x) to two dimensions (x,y). The Stokes parameters at the DIC microscope image plane are given by

$$S_{0}(x,y)=I(x,y,0\;\deg)+I(x,y,90\;\deg),$$ (16)

$$S_{1}(x,y)=I(x,y,0\;\deg)-I(x,y,90\;\deg),$$ (17)

$$S_{2}(x,y)=I(x,y,45\;\deg)-I(x,y,135\;\deg),$$ (18)

where I(x,y,φ) is the image intensity detected by a pixel behind a micropolarizer oriented at angle φ on the polarization camera and the $S_{i}(x,y)$ are the Stokes vector images. In this notation, Eq. (21) becomes

$$\theta (x,y)=F^{-1}[W_{1}(u)F\{\frac{1}{\Delta x}\;\tan^{-1}[\frac{-S_{2}(x,y)}{S_{1}(x,y)}]\}].$$ (19)

where Δx is the shear distance calculated in units of pixels, as in Eq. (15).

## 3 Evaluation of the Phase Accuracy

**Fig. 3:**
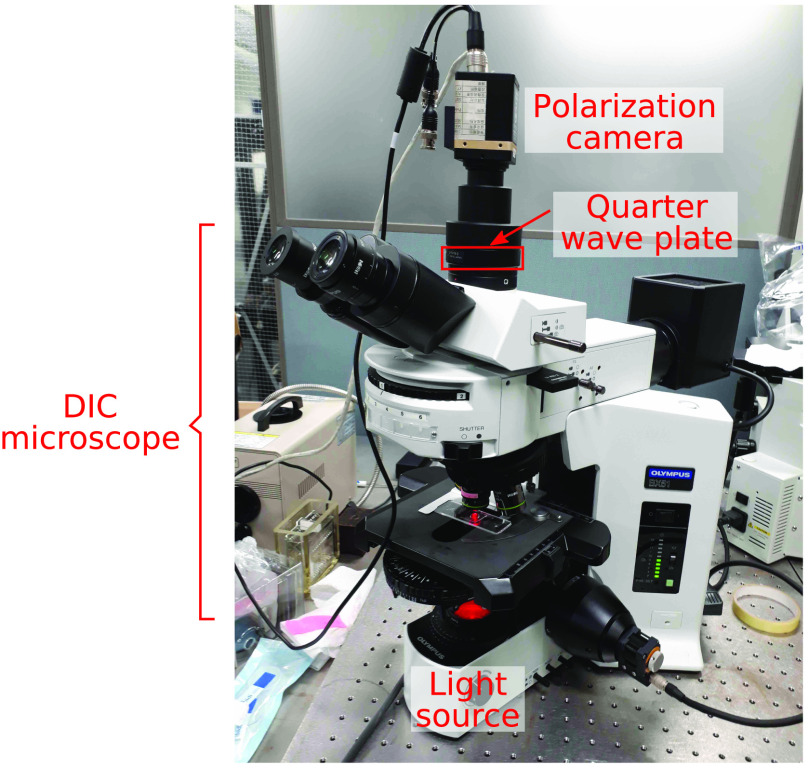
The DIC microscope with a polarization camera and quarter-wave plate.

Figure 3 shows a DIC microscope (Olympus BX51) using a 4-D Technology PolarCam (model V) polarization camera. The polarization camera pixel size is $7.5\times 7.5\;\mu m^{2}$, and the image size is 648×460  pixels. We use a halogen lamp light source, with a 640- to 950-nm spectral bandpass filter. The quarter-wave plate is not achromatic, and thus has a variation in retardance across the spectral range from 106 deg at 640 nm to 90 deg at the design wavelength of 745 nm to 69 deg at 950 nm.

In order to demonstrate the phase quantification of the system, we image a sample of resin beads (refractive index $n_{1}=1.57$) embedded in a specimen mounting agent medium (lacquer, refractive index $n_{2}=1.545$). The diameter of the resin beads is 3±0.05  μm and the magnification of the objective lens is 20× (NA=0.45).

Although the experiment only involved placing the scatterer between the condenser lens and the sample, the effect on the measurement should be similar to that if placed between the sample and objective lens. The main difference is that we should see a reduced scatter-induced attenuation when the scatterer is placed closer to the objective lens, due to a wider range of angles capable of collection through the objective. Unfortunately, the geometry of the microscope does not allow easy access to insert a scattering element between the sample and objective lens, so that the experiment only demonstrates scattering from one side of the sample.

**Fig. 4:**
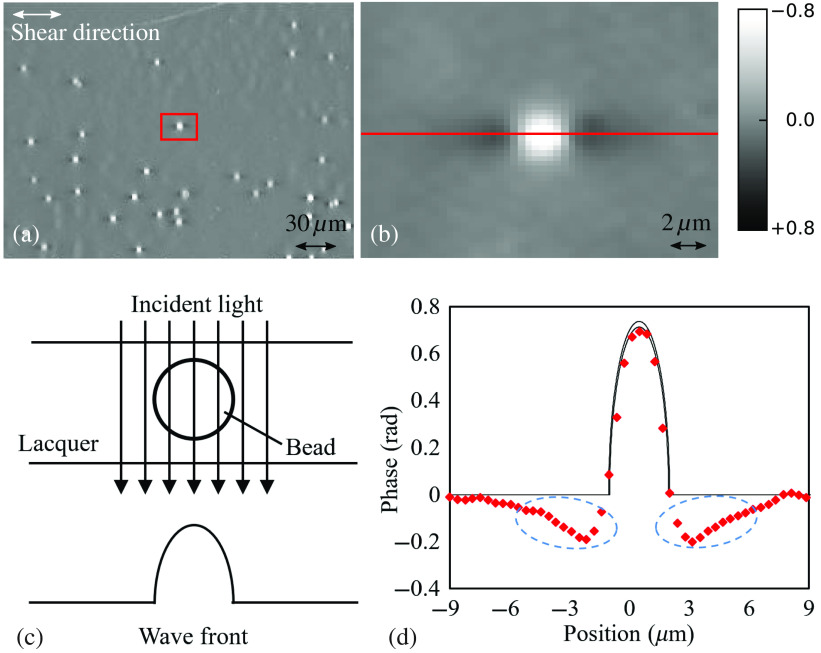
(a) The quantitative phase image of resin beads ($n_{1}=1.57$) embedded in lacquer ($n_{2}=1.545$). The magnification of the objective lens is 20× (NA=0.45). The image is sampled at 0.38  μm/pixel. (b) Enlarged view of the red box region indicated in (a). (c) The wave front of the transmitted light through a bead. (d) The cross section through the center of the resin bead [red line in (b)]. The two black curves give the theoretical value of the phase for bead diameters 2.95 and 3.05  μm.

Figure 4 shows the measurement results by the proposed method. Figure 4(a) shows the quantitative phase image of resin beads ($n_{1}=1.57$) embedded in lacquer ($n_{2}=1.545$). Figure 4(b) gives the enlarged view of the red box region indicated in (a). Figure 4(c) shows the wavefront of the transmitted light through a bead. Figure 4(d) compares the measured phase with the theoretical phase in the center of the resin bead [red line in Fig. 4(b)]. At the center of the bead, where the phase is large, the measured plots follow the theoretical curve. The artifact appears at both sides of the bead, as indicated by blue dashed ellipses in Fig. 4(d) because the light is deflected. Yu et al.^24^ showed that the light is deflected when passing through the transparent inclined surface, which affects the measurement accuracy.

Although the 640-to 950-nm bandpass limits the chromatic variation of the waveplate’s retardance, the residual variation of +16  deg to −21  deg from the nominal 90 deg value causes a small amount of error in the phase estimate—generally <5% when used with our light source and detector. If desired, this error can be further reduced by calibrating the waveplate and modifying the phase shift algorithm to incorporate the nonideal retardance.^25^

## 4 Influence of Scattering on the Phase

**Fig. 5:**
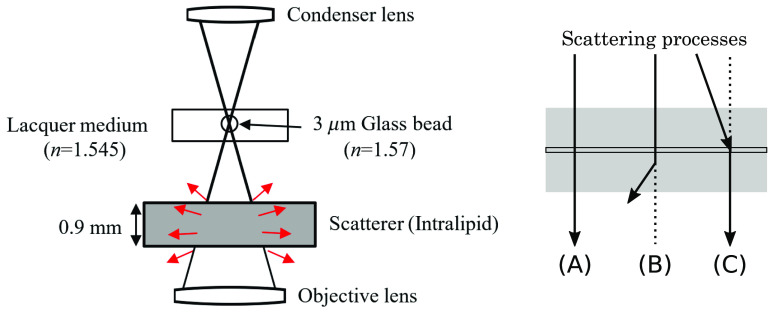
The experimental set up to demonstrate the influence of scattering on the phase measurement and a schematic showing the possible light propagation paths: (a) ballistic; (b) scatter loss; and (c) channel mixing.

For light passing through a singly scattering medium, we can classify the possible paths into three different categories (see Fig. 5):

(A) ballistic path (no scatter),
(B) scattering out of the light collection path, and
(C) light from a nearby path scattering into the ballistic path.

Although propagation path (B) simply causes a loss of signal, path (C) causes the phase acquired along an alternative path to mix with the phase acquired along the ballistic path. Since the scatter process in the (C) path tends to alter the light’s polarization state, the polarizer at the detector array is able to reduce the amplitude of (C) light according to the degree that its polarization state differs from the ballistic state.

For ballistic light, the measured polarization state and phase follow the development of Eqs. (1)–(19). For channel mixing light, however, the polarization state is largely scrambled, so that the measured light intensity at each polarization filter is independent of the input state and object phase. Thus, if we write the input light intensity as $I_{0}$, the ballistic component as $I_{A}$, the loss due to scattering as $I_{B}$, and the channel mixing component as $I_{C}$, then the ballistic component is simply $I_{A}=I_{0}-I_{B}$. This component gives the amplitude for the interference contrast [Eq. (4)]. The channel mixing component is depolarized and thus does not participate in the interference, so that the resulting light intensity measurement is simply a sum of $I_{C}$ with the intensity given in Eq. (19)^21^

$$I_{\mathrm{meas}}=I_{C}+I_{A}[\alpha_{1}^{2}\;\cos^{2}\;\varphi +\alpha_{2}^{2}\;\sin^{2}\;\varphi +2\alpha_{1}\alpha_{2}\;\cos\;\varphi \;\sin\;\varphi \;\cos(\Delta \theta +\beta )].$$ (20)

In order to quantify the influence of scattering on the quantitative phase estimate, we image a resin bead sample with a 0.9-mm-thick layer of intralipid placed between the sample and the objective lens (see Fig. 5). By varying the concentration of the intralipid, we can change the degree of scattering between the lens and the phase sample. We evaluate the phase measurement with the conventional method (using only two polarizer angles)^15^ and the proposed method (using all four polarizer angles).

With the conventional method, the sample phase gradient is calculated using an approximation given by

$$\Delta \theta (x)\approx \frac{I(x,45\;\deg)-I(x,135\;\deg)}{I(x,45\;\deg)+I(x,135\;\deg)}=-(\frac{I_{A}}{2I_{C}+I_{A}})(\frac{2\alpha_{1}\alpha_{2}}{{\alpha_{2}}^{2}+{\alpha_{1}}^{2}})\;\sin[\Delta \theta (x)],$$ (21)

where $\alpha_{1}$ and $\alpha_{2}$ are the amplitudes of the two interfering beams, respectively [see Eq. (2)]. The two factors in parentheses are present here but not in Eq. (4), though we can see that both factors approach 1 as the amount of scattering approaches zero. Thus, in the conventional algorithm, scattering will cause a reduction in the estimated phase. In the four-angle approach, however, the algorithm is able to compensate for scattering effects so that the only problem remaining is an increase in the noise, and a decrease in the ballistic fraction, with an increase in scatter.

**Fig. 6:**
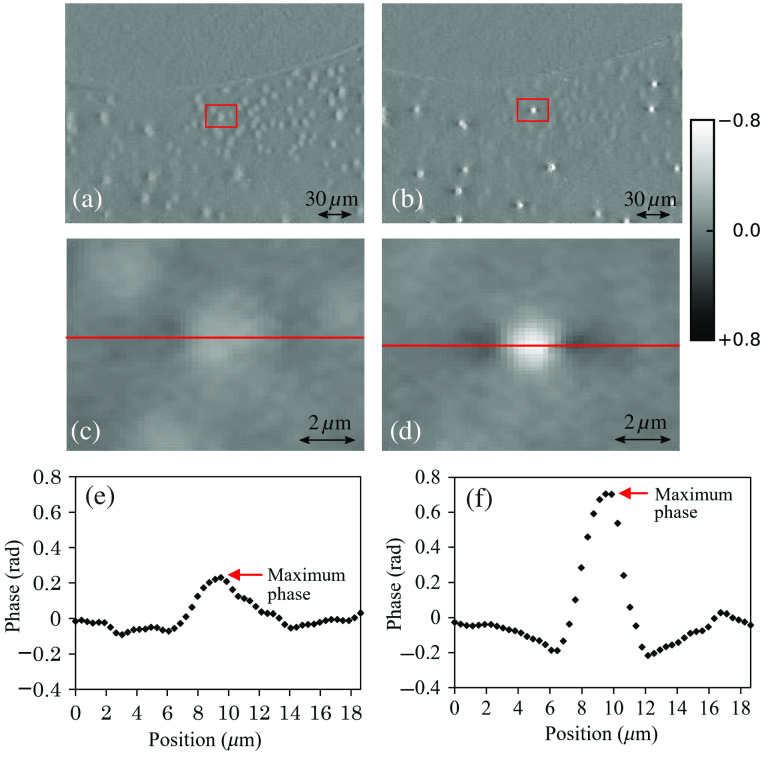
The quantitative phase of resin beads ($n_{1}=1.57$) embedded in lacquer ($n_{2}=1.545$) at the intralipid concentration of 2.0% where the phase is calculated using [(a), (c), and (e)] the conventional method and [(b), (d), and (f)] the proposed method. The magnification of the objective lens is 20× (NA=0.45). The image is sampled at 0.38  μm/pixel. Subimages (c) and (d) are enlarged images of the red squares indicated in (a) and (b), respectively. Subimages (e) and (f) are the cross sections through the centers of the glass beads [red lines of (c) and (d)].

Figure 6 shows the measurement results by the conventional method (shown on the left side of this figure) and the proposed method (shown on the right-hand side of this figure) at an intralipid concentration of 2.0%. Although imaging through nonscattering volumes (e.g., pure water) has no effect on the contrast, imaging through the scattering induced by the intralipid causes a loss of contrast in the conventional method. The proposed method, on the other hand, experiences only an increase in noise and a decrease in signal (due to a reduction in the ballistic fraction) in the presence of scattering, but does not experience a change in the mean of the estimated phase.

**Fig. 7:**
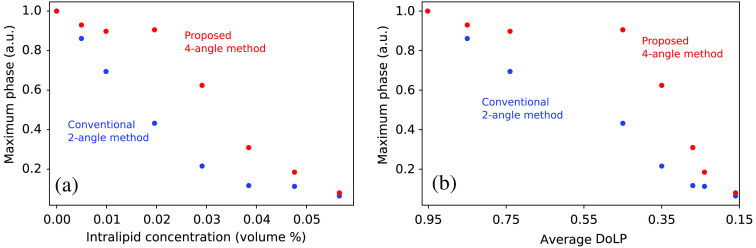
Influence of the scattering intensity of an intralipid sample on (a) the maximal phase and (b) the DoLP on the maximal phase. In both figures, the phase value is normalized to the value with no intralipid (water only sample).

Figure 7(a) shows the change of the phase measured at the center of the bead with increased scattering (intralipid concentration). We can see that the phase calculated with the conventional method decreases sharply with increase in scatter, whereas the phase calculated with the proposed method is more robust to scatter.

One way to quantify the severity of scatter is to measure the change in the degree of linear polarization (DoLP) of light transmitted by the sample. As the scatter increases, the ballistic photons retain their polarization state but the scattered photons do not, so DoLP is an estimate of the ballistic fraction. The DoLP is given by

$$\mathrm{DoLP}=\frac{\sqrt{{S_{1}}^{2}(x,y)+{S_{2}}^{2}(x,y)}}{S_{0}(x,y)}.$$ (22)

Figure 7(b) shows the change of the maximum phase, at the middle of a 2-μm glass bead sample, with the average DoLP in the image. Although the phase obtained with the conventional method rapidly decreases with reduced DoLP, the proposed method maintains the phase value for DoLP of ∼0.45 or more. Below DoLP=0.45, we see that scattering can induce errors of more than 10% in the phase estimate.

Because each sample presents different scattering behaviors, it is not easy to establish limits on the maximum depth in which accurate phase estimation is possible. However, if the scattering coefficient $\mu_{s}$ of the sample is known, then one can estimate the maximum depth from the result of Figure 7(b). For example, using established estimates of the intralipid scattering coefficient^26^ for the wavelength range of our measurements, we have

$$\mu_{s}(745\;\mathrm{nm})=325\;{\mathrm{mm}}^{-1}\;,\;\;\mu_{s}(1064\;\mathrm{nm})=181\;{\mathrm{mm}}^{-1}\;,$$

so that our 0.9-mm-thick intralipid sample introduces scattering-induced transmission of 0.42 and 0.78, respectively, when diluted to our 2% concentration. Taking the average of these two as a representative value over our spectral range gives a transmission of 0.60. Assuming that scattered light is unpolarized and ballistic light remains fully polarized, this represents a DoLP of 0.6. This is somewhat higher than our measured DoLP of 0.45, but considering the variations in intralipid properties, and difficulties of accurately quantifying such small samples, it provides rough agreement. As a result, users should be able to use DoLP as a proxy for the ballistic fraction.

## 5 Examples of Real-Time Phase Analysis

**Fig. 8:**
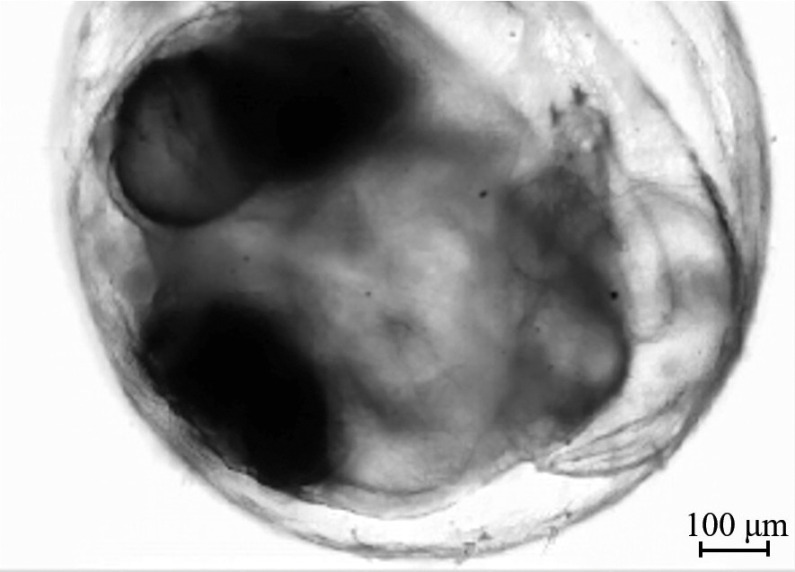
The intensity image of a living medaka egg used as the sample for real-time analysis. (This is the Stokes parameter $s_{0}$ image from the DIC microscope’s polarization camera.)

In order to demonstrate how the improved phase robustness allows us to quantify phase structures deeper within biological tissues, we perform real-time phase analysis of heart and blood motion within a living medaka egg (Fig. 8). The egg is ∼1  mm in diameter, and the sectioned image plane containing the heart appears roughly at the center of the egg, so that the phase image represents a section that is 500-μm deep inside the tissue surface.

**Fig. 9:**
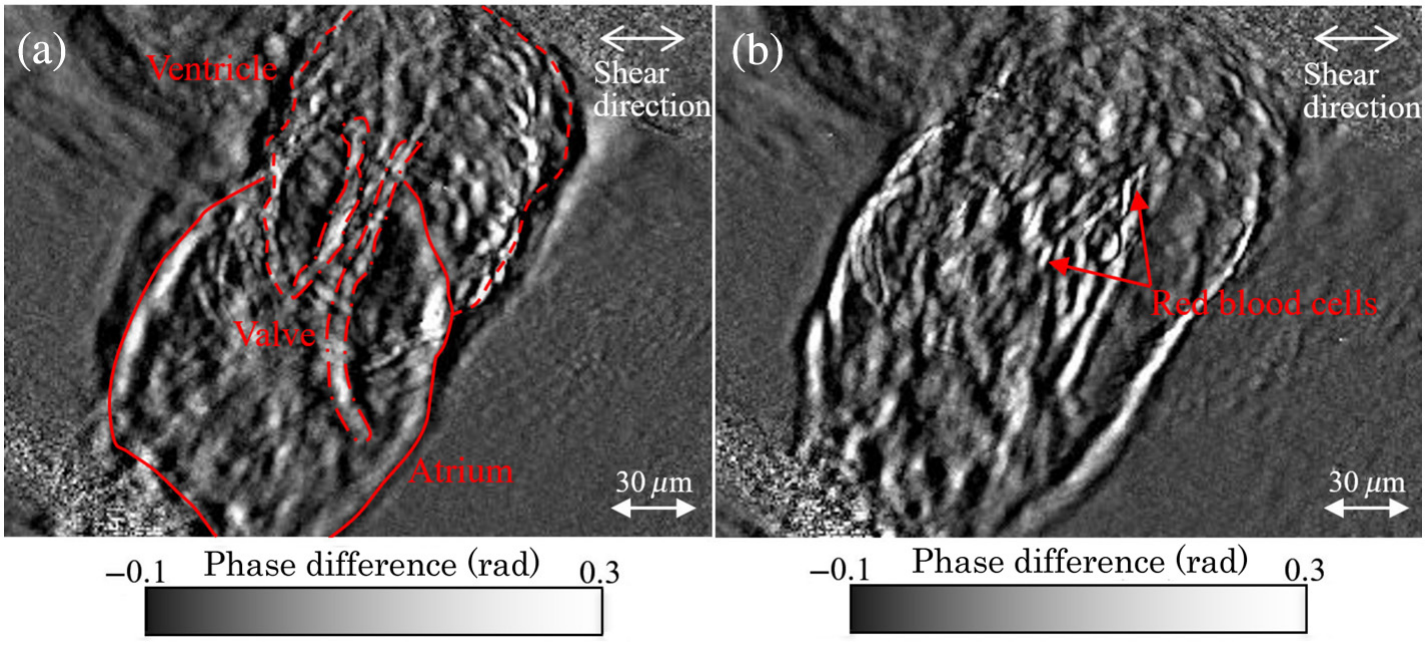
Two frames from a video of a living medaka heart in (a) contraction and (b) expansion phases, captured at 17 Hz frame rate. The white arrows at the top right of each image indicate the DIC shear direction. The average of all the images in the video is subtracted from each raw frame in order to remove clutter. The spatial sampling is 0.38  μm/pixel (Video S1, MPEG, 5.5 MB [URL: https://doi.org/10.1117/1.JBO.25.12.123703.1]).

Figure 9 shows an image taken from one frame of a 17-Hz video of the medaka’s heart movement and blood flow. In order to reduce clutter in the image, we take the average of all the images in the video sequence and subtract the mean phase image from each frame, so that the results are phase difference images rather than phase images. In the resulting video, we can observe the ventricle and atrium movement, the valve movement between the ventricle and atrium, and blood flow within the heart clearly despite the presence of scattering in the layers above and below the section plane.

**Fig. 10:**
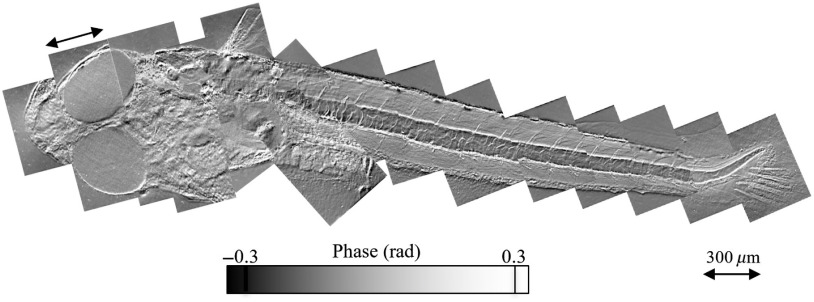
The movement within a horizontal section of a living recently hatched whole medaka captured at 17 Hz frame rate. The spatial sampling is 0.75  μm/pixel (Video S2, Moving Picture Experts Group (MPEG), 8.2 MB [URL: https://doi.org/10.1117/1.JBO.25.12.123703.2] and Video S3, MPEG, 8.0 MB [URL: https://doi.org/10.1117/1.JBO.25.12.123703.3]).

Next, we perform real-time phase analysis on the whole body of a living hatched medaka (Fig. 10). Because the 300-μm field of view of the microscope is too small to fit the entire 4-mm-length of the medaka’s body into one frame, we measure each individual field of view in real time, move the medaka, and then later stitch together all of the various fields of view into a single video mosaic. Figure 10 shows one frame from the resulting 17-Hz video measurement. In the result, we can observe the detailed structure of blood flow through the various parts of the medaka’s body: the mouth, heart, fins, and tail. Because the subimages were collected at different times, nonperiodic motion by the mouth, head, and fins are unsynchronized. However, despite these time gaps, the repetitive motion of blood through the medaka’s circulatory system gives the appearance of continuity, so that one can watch individual blood cells moving from veins behind the head to the tip of the tail fin, and then all the way back.

## 6 Conclusion

We have shown that by adding a quarter-wave plate to a DIC microscope using a polarization camera it is possible to improve quantitative phase accuracy in the presence of scattering. If only two orthogonal polarization angles are used to analyze the light transmitted through the DIC microscope, the estimated sample phase decreases with an increase in scattering (i.e., DoLP<1). The proposed method, on the other hand, maintains accurate phase measurement for deeper locations inside samples. Once the ballistic (unscattered) fraction of transmitted light drops below about 0.45, however, the loss in signal corrupts the phase estimate beyond the algorithm’s ability to compensate. Our imaging of the heart in a 1-mm-diameter medaka egg shows that the phase retains high contrast for imaging semitransparent biological tissues at depths of 500  μm and more.

The proposed method maintains compatibility with video measurement, allowing users to capture the phase motion of living samples at a rate limited only by the frame rate of the detector. Although the polarization camera used in our system operates at 17 Hz, more recent 5-megapixel polarization cameras can operate at speeds of 75 Hz.
